# Thermal imaging reveals audience-dependent effects during cooperation and competition in wild chimpanzees

**DOI:** 10.1038/s41598-022-07003-y

**Published:** 2022-02-22

**Authors:** Marion de Vevey, Alice Bouchard, Adrian Soldati, Klaus Zuberbühler

**Affiliations:** 1 Institute of Biology, University of Neuchâtel, Neuchâtel, Switzerland; 2 Budongo Conservation Field Station, Masindi, Uganda; 3 School of Psychology and Neuroscience, University of St Andrews, St Andrews, Scotland, UK

**Keywords:** Behavioural ecology, Social evolution, Evolutionary theory, Circulation

## Abstract

Accessing animal minds has remained a challenge since the beginnings of modern science. Here, we used a little-tried method, functional infrared thermal imaging, with wild chimpanzees during common social interactions. After removing confounds, we found that chimpanzees involved in competitive events had lower nose skin temperatures whereas those involved in cooperative events had higher temperatures, the latter more so in high- than low-ranking males. Temperatures associated with grooming were akin to those of cooperative events, except when males interacted with a non-reciprocating alpha male. In addition, we found multiple audience effects. Notably, the alpha male’s presence reduced positive effects associated with cooperation, whereas female presence buffered negative effects associated with competition. Copulation was perceived as competitive, especially during furtive mating when other males were absent. Overall, patterns suggest that chimpanzees categorise ordinary social events as cooperative or competitive and that these perceptions are moderated by specific audiences.

## Introduction

Like many other primates, chimpanzees live in social groups with defined membership, an adaptation that reduces predation risk and maximises both benefits from cooperation and costs from competition due to shared space and resources^1,2^. Most primate societies are characterised by kin- and alliance-based social networks that have both ecological and social functions^3–7^. In several primate species it has been demonstrated that access to kin and allies has direct fitness consequences in terms of longevity and reproductive success (e.g., baboons^8^; macaques^9^; bonobos^10^). The presence of kin and allies strengthens individuals during both competitive and cooperative events, such as resource-related conflicts, foraging or predator defence^11^. Compared to other primates, however, the chimpanzee social system has an added complexity due to its fission–fusion structure, a social adaptation that allows individuals to dynamically optimise the cost–benefit ratio of group living^1,2^.

Living in fission–fusion social systems is thought to be cognitively more demanding compared to stable groups, since individuals cannot monitor each other in real time but require mental bookkeeping of social data with regular updates^12^. These constantly changing social constellations thus require a psychological apparatus that can recognise and evaluate social events rapidly and accurately as well as prepare the system for appropriate decisions on whether to compete or to cooperate. Research on chimpanzee behaviour has correspondingly demonstrated high levels of social awareness and the presence of multiple audience effects, the latter being defined as the changes in the behaviour of individuals caused by the mere presence of other individuals^13^. For example, victims of aggression tend to avoid allies of the attacker in the future^14^ but exaggerate the nature of an attack in the presence of favourable audiences^15^. When discovering a snake, individuals are more likely to warn ignorant rather than knowledgeable audiences^16^, while females are less likely to reveal their sexual activity in the presence of other females than males^17^ and less likely to greet other males in the presence of the alpha male^18^. Each of these studies demonstrated the relatively complex ways in which individuals interacted socially, suggesting a cognitive apparatus capable of rapidly assessing challenging social constellations.

Although competition and cooperation are major organisational principles of animal behaviour, it has been surprisingly difficult to identify the underlying cognitive processes. Measures of neural, respirational, digestive or cardio-vascular activity have been used^19^, but for free-ranging animals such typically invasive methods are rarely applicable. One exception has been to assess event-related changes in cortisol and oxytocin metabolites from urine samples^20–22^, which has provided a new avenue into how chimpanzees and other animals perceive social interactions. Although results have been promising, there are lingering concerns about the accuracy of the assays and the relatively poor temporal resolution. In the present study, we used a little-tried, non-invasive method to assess internal states in wild animals: functional thermal infrared imaging. The procedure is based on capturing differences in the irradiation emitted from the skin surface, caused by differences in underlying blood flow, and convert measures into a two-dimensional thermal image^23,24^. The basic assumption is that different psychological states are associated with specific alterations of the blood flow underneath the skin of the face, caused by stimulation of sympathetic or parasympathetic nervous systems, which increases or decreases blood pressure in different areas. Vasoconstriction leads to a decrease of blood flow in the extremities, including the face (i.e., nose and ears), while vasodilatation leads to a temperature increase of the same areas^19^, presumably because blood flow is redirected towards the brain and muscles to prepare the system for rapid responses or to prevent blood loss in an eventual case of injury^25^. Thermal imaging has previously been calibrated with standard methods of measuring physiological activity, such as heart rate (electrocardiography), respiration (piezoelectric thorax stripes), skin conductance (galvanic skin response) or skin temperature (nasal thermistors)^24^. Furthermore, in wild chimpanzees the method has been used in two pilot studies investigating responses to vocalisations^26^ and differences in females’ reproductive states^27^.

Based on previous thermal imaging studies, it seems that stressful and socio-negative events are typically associated with lower nose temperatures, whereas for socio-positive events the patterns are unclear (see Supplementary Table S1). Thus, we predicted a lower temperature for individuals exposed to social threats (i.e., competitive situations), whereas we remained agnostic for cooperative interactions, which are more likely perceived as socio-positive events. Also, given the documented high-levels of social awareness in chimpanzees, we predicted temperature differences depending on audience compositions, whether it would be favourable (e.g., bond partners or kin) or hostile (e.g., dominant males), an effect which would be mediated by the social rank of the focal individual. To test these predictions, we collected and analysed facial thermal data of adult male chimpanzees engaged in a variety of ordinary social activities, including grooming and copulating, with different audience compositions.

## Results

### The perception of social events

We recorded *n* = 4143 thermal images of nine adult male chimpanzees of the Sonso community in Budongo Forest, Uganda (Supplementary Table S2). The pictures were taken during 13 different ordinary social event types (*n* = 1003 events, Table 1) from which we extracted nose temperatures (Supplementary Table S3).

**Table 1:** List of social events of interest with respective definitions.

Social event	Definition
Aggression	When another individual physically aggresses the focal individual through grasping, beating, or biting^30^. Although aggressions are usually non-lethal within a community, they can rarely be between different communities^39^
Solicitation for meat	When the focal individual is eating meat after a hunt and another chimpanzee begs to obtain some. This is usually accompanied by a “begging-reach” gesture of the beggar towards the recipient^58^
Patrolling	Group composed mostly by males that leave their territory and carry out a border patrol into the overlapping zone between their territory and that of a neighbouring community. Their behaviour changes into more cohesive and is accompanied by quiet movements and regular stops to listen intently^39^
Copulation	When the focal individual engages in reproductive sexual behaviour intercourse with a female. Copulation involves a high likelihood of an aggression from other individuals, mainly high-ranking males^17^
Display by another male	An individual threatening the focal individual by moving rapidly towards him, sometimes bipedally, while showing pilo-erection, exaggerated locomotion, branch shaking, throwing objects, stomping, slapping, or drumming^59^. This is done as a form of intimidation and to assert dominance^60^
Dominant arrival	When a dominant individual approaches the focal individual. This can elicit submissive behaviours, like pant grunting or leaving
Feeding	Eating provides useful and necessary resources to the individuals but can also be a social event for chimpanzees^61^. Indeed, eating can involve aggressions or competition for food and spend extensive time with other individuals
Female inspection	A non-reproductive socio-sexual behaviour consisting of touching and smelling the anogenital region of females. Inspection happens more often during genital swelling, and decreases as the presumed day of ovulation approaches^62^
Solicitation from a female	Female solicitation consists of a female presenting her anogenital region towards a specific male, mostly during the period of genital swelling^62^
Grooming	While grooming has primarily a hygienic purpose, in most primate species it also strengthen the social bonds between individuals^28^. There are three types of social grooming behaviours considered in this study: grooming given, grooming received, and mutual grooming
Hearing screams	When the focal individual is exposed to the screaming vocalisation of another member of the community. It only considered events where the human observers were able to hear the screams
Playing	Playing has an important role in primate development since it combines cooperation, communication and learning^29^. It elicits both spontaneous and replicated laughter in chimpanzees^63^
Snake encounter	When the focal individual sees a snake model on the ground. A plastic snake was put on the way of the focal individual when being alone in the party and later removed when out of sight of the focal individual following the protocol adopted in previous snake presentation experiments conducted in the same community^64^. Thermal pictures were taken when other individuals joined the focal individual after the snake presentation to consider its social aspect
Baseline	The focal individual is resting for at least 10 min on the same location without moving or engaging in social activities. Thermal pictures taken during this activity serve as reference in the analyses

**Figure 1:**
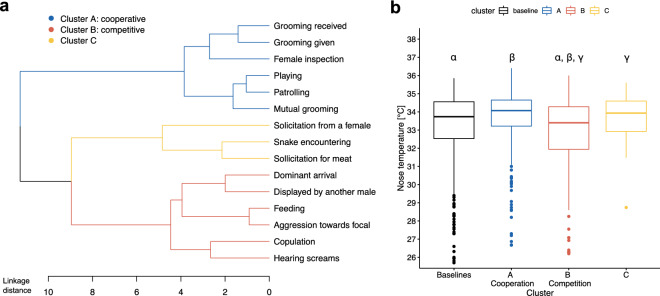
Results of cluster analysis of the events based on the nose temperature pattern. (**a**) Cluster analysis of events based on (**b**). Recorded nose temperatures during these events (n = 1003). The analysis revealed a segregation between cooperative (in blue, cluster A) and competitive events (in red, cluster B), as well as a cluster comprising events involving both cooperative and competitive aspects (in yellow, cluster C). The analyses showed differences between baselines and competitive events (‘α’, *p* = 0.056), between cooperative and competitive events (‘β’, *p* < 0.001) and between competitive events and cluster C (‘γ’, *p* = 0.064). The recorded nose temperatures for each event are shown in Supplementary Fig. S1.

We first carried out a global assessment of how different social events affected nose temperatures. To do so, we performed a hierarchical cluster analysis and found the best fit to consist of three clusters (Fig. 1a). Cluster A (blue), associated with higher nose temperatures, included the following six social events: grooming given, grooming received, mutual grooming, playing, patrolling, and female inspection. All six events can broadly be considered cooperative as they operate towards strengthening social relations (e.g., grooming^20,28^; playing^29^; patrolling^21^). Cluster B (red), associated with lower nose temperature, included the following six social events: dominant male arrival, display by another male, copulations, hearing screams, aggression and feeding in groups. All six events can be classified as competitive, either due to aggression (i.e., being aggressed^30^, hearing screams^31^, display by another male^32^) or due to high potential for aggression (i.e., dominant arrival^33^, feeding^30^, copulation^34^). Cluster C (yellow), associated with temperatures similar to baseline, included three events: solicitations for meat, solicitation for sex, and snake encountering. The commonality of these three situations is that subjects are being coerced into a cooperative role, either to hand over meat, to engage in sexual activity or to signal a danger to others.

We then compared the nose temperatures of the three clusters with each other and with a baseline (i.e., resting) and found significant differences (‘cluster model’, Anova: *χ*^2^ (3) = 19.027, *p* < 0.001; n = 1003; Fig. 1b). Post-hoc pairwise comparisons revealed that nose temperatures during competitive events (cluster B: mean = 32.8 ± 2.10 SD) were significantly lower than during cooperative events (cluster A: mean = 33.6 ± 1.66 SD; *t* (852) = 4.06, *p* < 0.001). Intermediate events (cluster C: mean = 33.6 ± 1.45 SD) were near-significantly different from competitive events (*t* (850) = − 2.48, *p* = 0.064), but not significantly different from cooperative events (*t* (850) = − 0.83, *p* = 0.843). The events from the baseline (mean = 33.1 ± 2.16 SD) showed a near significantly higher nose temperature than the cluster B events (*t* (850) = 2.53, *p* = 0.056) but no significant differences with clusters A and C (|*t*_s_(851)|≤ 1.87, *p*_s_ ≥ 0.240).

### Effects of audience composition

In a next set of analyses, we investigated how audience composition affected subjects’ perception of social events in both cooperative and competitive situations.

#### Cooperative social events

**Figure 2:**
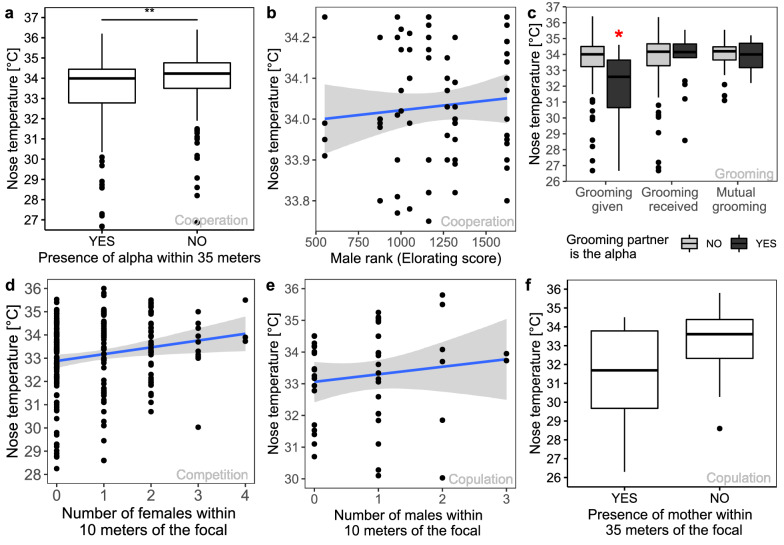
Thermal response to daily events considering social variables. Nose temperatures depending on (**a**). The presence or absence of the alpha male within 35 m during cooperative events (**p < 0.01) and (**b**) on the dominance rank of the focal individual during cooperative events (n = 339). (**c**) Nose temperatures depending on the type of grooming and if the grooming partner was the alpha male or not. The event when the focal individual is grooming the alpha without reciprocation is associated with lower nose temperatures compared to all other events (*p < 0.05) (n = 287). (**d**) Nose temperatures as a function of the number of females within 10 m during competitive events (n = 241). Nose temperatures depending on (**e**). The number of males within 10 m during copulation (n = 51) and (**f**) the presence of the focal individual’s mother within 35 m during copulation (n = 51). Whiskers show values within 1.5-fold of the interquartile range. Dots indicate individual values.

For cooperative events (cluster A; n = 392), we found that the best model to explain nose temperature variation contained two variables: the presence of the alpha male and the subject’s own rank (‘cooperation model’, LRT: χ^2^ (2) = 9.82, *p* = 0.007; Supplementary Table S4). First, nose temperatures were lower in the presence of the alpha male (presence: M = 33.40, SD = 1.83; absence: M = 33.90, SD = 1.44; Anova: χ^2^ (1) = 9.69, *p* = 0.002; Fig. 2a), suggesting that his presence reduced the overall positive perception of cooperation. Second, nose temperatures were generally higher in high- rather than low-ranking males, suggesting that high-ranking individuals perceive cooperative events more positively than low-ranking males (Anova: χ^2^ (1) = 2.35, *p* = 0.125; Fig. 2b). Even if this effect was not significant, it improved the fit of the model in the model selection procedure.

**Figure 3:**
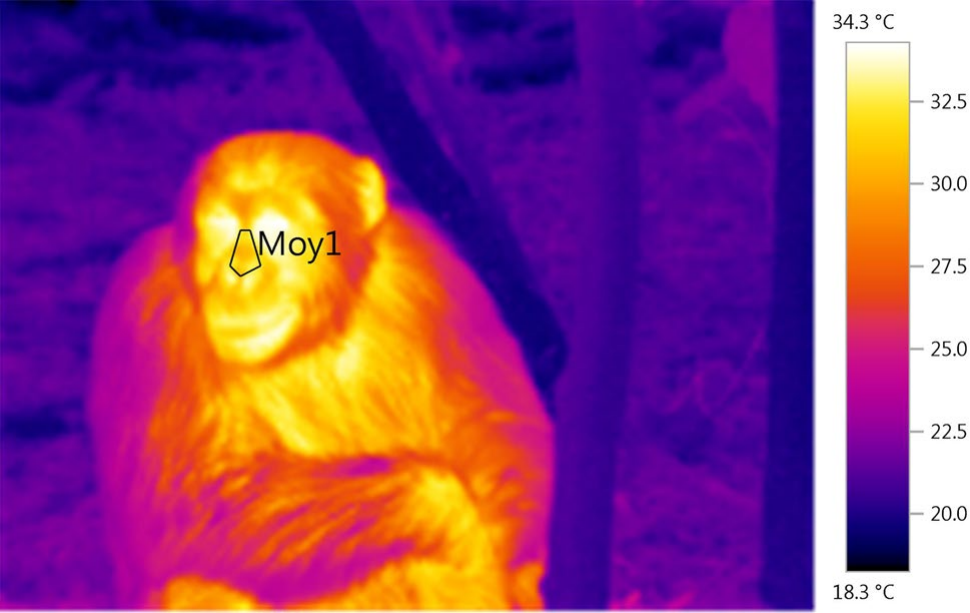
A thermal picture of a chimpanzee taken after the arrival of a dominant male. The black contour labelled "Moy1" represents the region of interest.

We then further investigated the variation of nose temperatures during grooming events (n = 287). The best model included the interaction between the subject’s role during grooming (given, received, or mutual) and whether the grooming partner was the alpha male (‘grooming model’, LRT: χ^2^ (5) = 21.68, *p* < 0.001; Supplementary Table S5). There was a significant effect of the social role during grooming (χ^2^ (2) = 7.52, *p* = 0.023). When grooming was given, nose temperatures were significantly lower than when receiving grooming or when grooming was mutual (given: M = 33.44, SD = 1.75; received: M = 33.61, SD = 1.85; mutual: M = 33.99, SD = 0.97; post hoc comparisons: given vs received: *t*(232) = − 3.26, *p* = 0.004; given vs mutual: *t*(231) = 3.55, *p* = 0.001; received vs mutual: *t*(232) = − 0.51, *p* = 0.869). When the grooming partner was the alpha male, the nose temperature of the focal individual was significantly lower (M = 33.3, SD = 1.81) than when grooming with other males (M = 33.7, SD = 1.64) (χ^2^ (1) = 7.38, *p* = 0.007). The interaction of these variables was also significant (χ^2^ (2) = 10.08, *p* = 0.006; Fig. 2c). Post hoc comparisons showed that nose temperatures were significantly lower when grooming the alpha without reciprocation compared to grooming any other male or in other social roles (|*t*_*s*_| ≥ 3.02, *p*_*s*_ ≤ 0.033). All other contrasts were not statistically significant (|*t*_*s*_| ≤ 1.91, *p*_*s*_ ≥ 0.398).

#### Competitive social events

For competitive events (cluster B; n = 241), the best model revealed that the number of females within 10 m was significantly related to nose temperature (‘competition model’, LRT: χ^2^ (1) = 5.16, *p* = 0.023; Supplementary Table S6). Specifically, the nose temperature of focal males was higher when more females were present (Anova: χ^2^ (1) = 5.01, *p* = 0.025, Fig. 2d), suggesting that females had a buffering effect during competitive interactions.

Finally, we explored temperature variations following copulation events (n = 51), which clustered in the competitive group. The best model revealed an effect of the number of males within 10 m and, to a lesser degree, the presence of the mother within 35 m (‘copulation model’, LRT: χ^2^ (2) = 7.79, *p* = 0.020; Supplementary Table S7). The nose temperature of the subject was higher when there were more males in the audience (Anova: χ^2^ (1) = 5.59, *p* = 0.018; Fig. 2e), suggesting that the competitive aspect of copulation is enhanced when there are fewer males in proximity. The presence of mothers had the opposite effect, i.e., temperatures were lower, albeit only near significantly so (present: M = 31.2, SD = 2.90; absent: M = 33.3, SD = 1.59; Anova: χ^2^ (1) = 3.19, *p* = 0.074; Fig. 2f), possibly due to strong female-female competition in this group^35^. However, only five of nine focal males had a living mother in the community (see Supplementary Fig. S2), which prevented us from drawing any firm conclusions.

## Discussion

We collected infrared thermal data from wild chimpanzees engaged in daily social activities and found that peri-nasal skin temperature varied in systematic ways, depending both on the type of social event and on the audience composition. Events deemed as competitive (aggression, copulations, dominant arrival, display by another male, hearing screams and feeding with others) were associated with lower temperatures, probably due to a general increase in stress levels (see comparable effects in humans^36^, macaque monkeys^37^ and common marmosets^38^), whereas events regarded as cooperative (grooming^20,28^, playing^29^, patrolling^21,39^ and inspecting females) were associated with higher temperatures, probably due a general decrease in stress levels^40,41^. These patterns are consistent with previous findings by Brügger and colleagues^42^, who found nose temperature increases in marmosets exposed to cooperative vocalizations and decreases in response to aggressive vocalisations, similar to what has been reported in humans^43^. Of particular interest were the observed intermediate temperatures when chimpanzees were solicited for cooperation, such as to hand over food, being solicited for sex (young females often solicit although they are not interesting for males^44^) and encountering a snake, which require lengthy guarding and warning others^16^. Further studies are necessary to clarify the interplay between the cooperative and competitive aspects during those complex events. Responses to cooperative events were generally mediated by the subject’s own dominance rank, with high-ranking individuals showing stronger responses than low-ranking individuals (i.e., higher nose temperatures), possibly due to different cooperative abilities (e.g., during mate guarding^45^).

As already demonstrated by various behavioural studies, chimpanzees can be strongly influenced by specific audiences in how they respond to social events. First, and somewhat unexpectedly, we did not find any effect of the presence of bond partners, neither during cooperative nor competitive events. However, our results showed that the presence of the alpha male prevented the manifestation of higher temperatures typically associated with cooperative events, possibly due to fear of his aggression. Grooming events were associated with higher temperatures, reflecting the cooperative nature of the behaviour. This finding was unsurprising and in line with previous studies revealing that grooming strengthens bonding^28^ and is correlated with oxytocin release^20^, a hormone linked with socio-positive behaviour and a buffer against stress in humans^46^. Unexpectedly, however, when subjects groomed the alpha male and the later did not reciprocate, temperatures were lower, potentially again indicating an increase in the level of stress, despite the overall positive nature of grooming.

Audience effects were also seen during competitive interactions. When a male chimpanzee faced a competitive event, the number of females nearby had a buffering positive effect, suggesting that relationships with females have an underestimated positive impact on male chimpanzees. Regarding copulation, we replicated earlier findings in bonobos, which showed that mating was perceived as something competitive^47^. Here, the number of males in the audience (< 10 m) also had a moderating effect, but again in a somewhat unexpected way. Nose temperatures were lower when fewer males were present, potentially indicating an increase in stress levels. This is probably due to increased likelihood of aggressions when copulation is hidden: when more males are present, the copulation is more overt and accepted, and thus represents a more predictable and less stressful event. Similarly, when mating with low-ranking males, chimpanzee females typically refrain from producing copulation calls, presumably to remain concealed and so avoid attracting attention and eventual aggression^17^. Second, the presence of a focal male’s mother also appeared to have a negative effect, which is difficult to interpret but suggests that mother-son relationships have an impact on how males interact with other females^48^. Due to small sample size and large variation between subjects, we can only speculate regarding this effect.

In sum, our study showed that the physiology of chimpanzees is strongly adapted to the ubiquitous cooperation-competition dynamic in their life, allowing individuals to assess and react to complex social constellations in clear-cut ways. It is tempting to interpret the reported temperature patterns as reflections of underlying emotional states^38,49,50^. In humans, two main functional classes of emotions have been discriminated^50^: protective (to combat danger, threat or loss) and nurturing (to secure resources^51^), which present some parallels to the cooperative and competitive social events we found to play a role in our study. Crucially, in humans it is not the event itself that triggers emotions but the perception or anticipation of the event^50^. This again parallels the audience effects seen in our study, since the presence of specific individuals in the audience can considerably change the likely outcome of an event. Further investigations to clarify the parallels and distinctions between human emotional categories and their correspondence in non-human primates are necessary.

Our study suggested that chimpanzees cognitively categorise most social events as either cooperative or competitive, but that these perceptions are moderated by audience composition, which may explain some inconsistent results in previous studies. Indeed, previous researches hypothesised before that facial temperature changes were linked with arousal^38,49^, valence^43^, or cognitive workload^52^.

Overall, our study further also reiterates the validity and value of functional thermal imaging as an efficient tool to infer mental states in wild chimpanzees and other animals in a direct and non-invasive way. Obtaining simultaneous physiological measurements (especially hormones) would be a desirable next step and thermal responses in other relevant body parts could also be investigated.

To conclude, our study led to three major findings. Firstly, we found that wild chimpanzees’ nose temperatures varied systematically between social events according to their competitive and cooperative nature, seemingly a reflection of the negative or positive perceptions of such events, thus suggesting that thermal imaging could serve as a “window to the mind” of this species. Secondly, the variations in nose temperatures revealed a cognitive categorisation of the different events according to their competitive and cooperative aspects, which corroborates the importance of the cooperation-competition dynamic in the cognition of chimpanzees. Thirdly, it revealed that audience composition affects the physiological responses of chimpanzees to different events, thus highlighting the crucial role of social cognition in their perception of these events. It remains to be tested if human psychological states are similarly affected by cooperation-competition dynamics. In sum, infrared thermal imaging can be a reliable method to investigate chimpanzee psychology and provides a novel tool for studying the evolution of cognitive capacities in primates.

## Methods

### Study subjects

Nine adult male chimpanzees (*Pan troglodytes schweinfurthii*) of the Sonso community of Budongo Forest, Uganda, were observed from October 2019 to March 2020 (Supplementary Table S2). The Sonso community has been studied continuously since 1990 and is habituated to human observers^53^. At the time of the study, the group comprised 71 individuals, including 9 adult males and 31 adult females (Supplementary Table S3). We only followed males since they are more often engaged in social interactions than females^3^ both in cooperative and competitive ways^4^. Ethics approval of research was granted by the Ugandan wildlife authority (SS351ES) and the Uganda National Council of Science and Technology (UWA/COD/96/05). The study was conducted following the ASAB guidelines (10.1016/j.anbehav.2019.11.002).

### Data collection

On a given day, a focal individual was selected and followed continuously from 07:00 to 16:30. Whenever a social event of interest (as shown on Table 1) occurred during focal follows, thermal images of the subject’s face were taken at a distance of approximatively 7 m (range: 7–15 m), provided there was a direct and unobstructed line of sight and no direct sunlight on the subject. The thermal pictures were taken with a Testo *881-2* thermal imager with a spectral range of 8–14 µm, a thermal sensitivity smaller than 50 mK at 30 °C and an emissivity of 0.98, a value detected for human skin^54^ and used for chimpanzee’s thermal imaging^26^. All pictures were taken using a telephoto lens 9° × 7°/0.5 m, with a resolution of 1.0 mrad. Since previous research showed thermal changes occur after the first 15 s following an event^55^, we took as many pictures as possible between 15 s and up to 300 s after the event of interest. The number of thermal pictures varied depending on the ability to have an unobstructed access to the face of the chimpanzee within the timeframe (range 1–13 pictures). Only events during which at least one other individual was present within a radius of 35 m from the focal animal (i.e., party^56^) were considered.

For every event during which we collected thermal images, we first recorded the identity and the activity of the focal subject (as in Table 1), as well as the number and identity of individuals present within 10 m (i.e., close proximity) and in the party (i.e., within 35 m). The movement of the chimpanzee preceding the event was also recorded since it can cause a temperature decrease in chimpanzees’ perinasal skin area^57^. We distinguished three categories: *no movement* (subject did not move within the previous five minutes), *steps* (subject did < 20 steps within the previous five minutes), and *travel* (subject did > 20 steps within the previous five minutes). Finally, we recorded the distance between the animal and the camera by estimation (after training with a laser meter), the ambient temperature and humidity using a digital thermometer and hygrometer (HTC-1 LCD).

### Coding

For each thermal picture, we extracted the mean temperature within a 5 angles diamond shape which included the peri-nasal area (Fig. 3). The two top angles were situated on both sides of the nose, at the level of the eyes, whereas the three bottom ones included the bottom of the nose. We made sure to exclude the nostrils from the shape to avoid incorporating skin temperature changes caused by the air flow entering and exiting the nose. This shape was hand-made using the built-in polygonal feature of the software *Testo IRSoft* (Version 4).

Chimpanzees rarely orient their faces directly at human observers, causing considerable variation in the angle between the head orientation and head-camera axis, which can induce temperature variation^65^. We thus estimated the angle at which the face of the animal was recorded in the picture within seven categories (Supplementary Fig. S3) and noted that this variation appeared in the related nose temperatures extracted from our dataset (Supplementary Fig. S7c). Therefore, we adjusted the recorded temperatures according to the angle at which the picture was taken. For each event during which we took pictures with a different angle than the *facing* angle (i.e., difference of angle between the camera and the looking direction of the chimpanzee of less than 45°), we corrected the measurement using the temperatures recorded at a *facing* angle and the ‘missMDA’ R package version 1.18^66^, which uses a regularized iterative principal component analysis (PCA) method. This resulted in 479 values corrected (i.e., = 46.06% of the dataset). This correction was viable considering the high correlations between each angle and the angle of reference (i.e., *facing*) (see Supplementary Fig. S4). We then attributed to each event the mean calculated using the nose temperatures extracted from all the pictures taken after the correspondent event.

### Definition of social bonds

The social bonds between all the chimpanzees of the Sonso community were measured using a Composite Relationship Index (CRI), a dyadic index which allows to take into account both socio-positive and socio-negative interactions^14,16,20^. Derived from Crockford et al.^16^, the CRI was calculated from long term data recorded by field assistants during the 6 months prior to this study (beginning of April 2019 to end of September 2019), as follows:

$$CRI = \frac{{{\text{Grooming}}_{focal - j} + {\text{ Resting within }}5{\text{ m proximity}}_{focal - j} - 5 \cdot {\text{Aggressions}}_{focal - j} }}{{\text{Total focal occurrences}}}$$

With *Grooming*_*focal−j*_ being the number of 15 min occurrences during which the focal individual was observed grooming, being groomed or mutually grooming the individual j; *Resting within 5 m proximity*_*focal−j*_ is the number of 15 min occurrences during which the individual j was observed resting within 5 m of the focal individual; *Total focal occurrences* is the number of 15 min occurrences that the focal individual was observed during those 6 months. *Agressions*_*focal−j*_ is the number of aggressions (i.e., displays, chases, threats and severe aggressions) that the focal individual participated in (being either the aggressor or the victim) during his observation. The number of severe aggressions were multiplied by two in order to give them more weight compared to other types of aggressions. The total aggression score was multiplied by five to consider the difference between the number of aggressive occurrences, calculated by events, and the grooming and resting within 5 m occurrences, calculated by 15 min occurrences, the later taking too much weight and overcoming the aggression rates if no correction is applied. Positive CRI scores indicated socio-positive relationships whereas negative CRI scores designated socio-negative relationships. For each focal individual, the three individuals with whom he shared the highest CRI scores (above zero) were considered as bond partners while the three lowest CRI scores (below zero) were considered as non-bond partners (Supplementary Fig. S5).

### Dominance hierarchy

The Elo-rating method was used to calculate the dominance hierarchy between the adult males based on a continuous update rating of interactions between them^67^. The calculation took into account three types of aggressions (i.e., severe aggressions, chases, and threats) as well as pant grunts, a unidirectional submissive vocalization^68^. To have an accurate estimation of the hierarchy at the beginning of the study, we used long-term data collected by the field assistants including a period of 6 months preceding data collection to the actual study period (from 02 April 2019 to 26 sept 2019). Given that the hierarchy was stable throughout the study period, we only used the final Elo-rating score (i.e., at the end of the study period) for each male (Supplementary Fig. S6). These Elo-rating scores were standardised for the ensuing statistical analysis.

### Statistical analysis

#### Baseline calibration

To test the potential effect of confounding variables on facial temperature, we ran linear mixed models (LMMs) with Gaussian error structure and identity link function, on the data collected during resting (i.e., baseline; n = 392). For each potential confounding variable, i.e., distance to the focal individual (m), ambient humidity (%), ambient temperature (°C) and subject’s prior movement (no movement/steps/travel), we ran a model entering the variable as a fixed effect with the log-corrected nose temperature as the dependent variable and the identity of the subject as a random factor. The results of these analyses showed that the nose temperature was significantly affected by the ambient temperature (Anova: *χ*^2^ (1) = 75.963, *p* < 0.001; Supplementary Fig. S7a) and the subject’s prior movement (Anova: *χ*^2^ (2) = 39.843, *p* < 0.001; Supplementary Fig. S7b), but not by the distance to the focal individual (Anova: *χ*^2^ (1) = 0.002, *p* = 0.966) nor the ambient humidity (Anova: *χ*^2^ (1) = 0.890, *p* = 0.346). We thus controlled for the ambient temperature and the subject’s prior movement in the ensuing model analyses. For clearer interpretation of the parameters, we centred the ambient temperature around its mean.

#### Cluster analysis

To determine if the different events of interest we considered in this study could be categorised depending on the resulting face temperatures, we conducted a hierarchical clustering analysis of these events based on their associated nose temperatures with the R function *hclust* of the R package ‘cluster’ version 2.1.2^69^, with the Ward’s minimum variance method (correlation of 0.48 with the Ward's method compared to 0.38 with the single method). The number of adequate clusters was chosen based on silhouette measures, showing that 3 groups was the best fit for all events, with all silhouette width being positive but < 1 (See Supplementary Fig. S8).

#### Cluster comparison

We then compared the different clusters among themselves and with the baseline temperatures by running a LMM (i.e., ‘cluster model’) with Gaussian error structure and identity link function, with the log-corrected nose temperature as the dependent variable and the cluster (A/B/C/Baseline) as a fixed effect. We also entered the identity of the focal individual and his previous movement as random factors and the ambient temperature as a control variable.

#### Audience effect

The temperature variations among cooperative (i.e., cluster A) and competitive events (i.e., cluster B) were then investigated using a model selection approach to determine which social parameters influenced these variations. We ran a model on the data from cluster A (i.e., ‘cooperation model’) and another model on the data from cluster B (i.e., ‘competition model’), to investigate the physiological responses to cooperative and competitive events, respectively. Finally, we further inspected the effect of social parameters on temperature variation during specific cooperative or competitive events, by running one model on the data collected during grooming interactions (i.e., ‘grooming model’) and one model on the data collected during copulations (‘copulation model’). We first built LMMs with Gaussian error structure and identity link function, with the log-corrected nose temperature as the dependent variable. For each model, the identity of the focal individual and his previous movement were used as random factors and the centred ambient temperature was entered as a control variable. We considered the following fixed effects in every model: presence of the alpha male within 35 m (yes/no) and within 10 m of the focal individual (yes/no); presence of the focal individual’s mother within 35 m (yes/no) and within 10 m of the focal individual (yes/no); number of females and number of males within 35 m and within 10 m of the focal individual; presence of a bond partner within 35 m (yes/no) and within 10 m of the focal individual (yes/no); presence of a non-bond partner within 35 m (yes/no) and within 10 m of the focal individual (yes/no); the nearest neighbour of the focal individual is a bond partner (yes/no); a non-bond partner (yes/no); a higher-ranking male (yes/no); the dominance rank of the subject (Elo-rating). For the ‘grooming model’, we also considered if the grooming partner was: a bond partner (yes/no), a non-bond partner (yes/no), the alpha male (yes/no), or a higher-ranking male than the focal individual (yes/no).

Then, for each model, we determined which combination of the above variables best fitted the data by using an automated model selection method based on Akaike’s information criterion with a correction for small sample sizes (AICc), with the *dredge* function of the ‘MuMIn’ R package version 1.43.17^70^.

Finally, we compared the selected models to their corresponding null models (i.e., including only the random factors and the control variable) with a likelihood ratio test (LRT), using the function *lrtest* of the ‘lmtest’ R package version 0.9-38^71^. We checked the assumptions of all the models calculating variance inflation factors using the *vif* and *gvif* functions of the ‘performance’ R package^72^. We also checked residuals for homogeneity and normality inspecting fitted vs. residual plots and quantile–quantile plots for the residuals^73^. All models were built using the *lmer* function of the ‘lme4’ R package version 1.1-26^74^, and the significance of the tested variables were established using the *Anova* function of the ‘car’ R package version 3.0–10^75^. We computed post hoc pairwise contrasts when necessary, using the ‘emmeans’ R package version 1.5.4^76^. Analyses were carried in R version 4.0.2^77^.

## Supplementary Information

Supplementary Information.

## Data Availability

All data generated or analysed during this study can be found by following this link: https://doi.org/10.6084/m9.figshare.17136965.v1.
