# Supplementary material for: Thermal imaging reveals audience-dependent effects during cooperation and competition in wild chimpanzees
Source: Sci Rep. 2022 Feb 22;12:2972. doi: 10.1038/s41598-022-07003-y (PMC8863809; doi:10.1038/s41598-022-07003-y)
Supplement: Supplementary file 1 — Supplementary Information. [file 41598_2022_7003_MOESM1_ESM.pdf]

Thermal imaging reveals audience-dependent effects during cooperation and competition in wild chimpanzees

SUPPLEMENTARY MATERIALS

Marion de Vevey [1,2*], Alice Bouchard[1,2], Adrian Soldati[1,2,3], Klaus Zuberbühler [1,2,3]

[1] Institute of Biology, University of Neuchâtel, Neuchâtel, Switzerland;

[2] Budongo Conservation Field Station, Masindi, Uganda;

[3] School of Psychology and Neuroscience, University of St Andrews, St Andrews, Scotland (UK).

*Corresponding author. Email: m.devevey@outlook.com.

SUPPLEMENTARY FIGURES

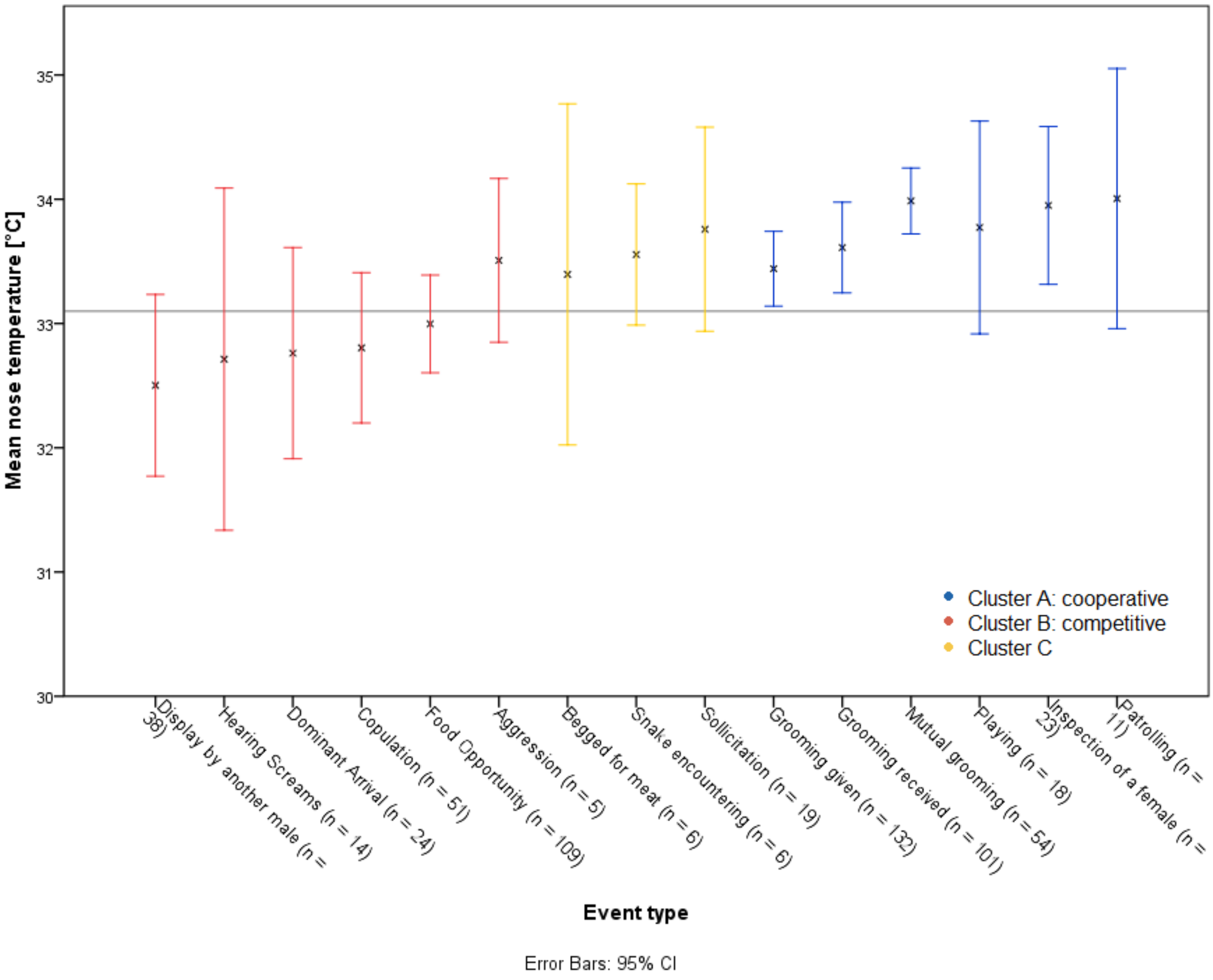

Supplementary Figure S1. Mean nose temperatures across all events. Cluster groups are represented by different colours: cluster A (blue) are best described as cooperative events, cluster B (red) best as competitive events, cluster C (yellow) involving both aspects. The horizontal line represents the baseline nose temperature. Error bars represent 95% confidence intervals (n = 1003*)*.

Focal ID	Number of recorded copulations with mother present	Number of recorded copulations with mother absent
**HW**	1	9
**JS**	2	2
**KT**	1	1
**MB**	3	7
**MS**	4	3

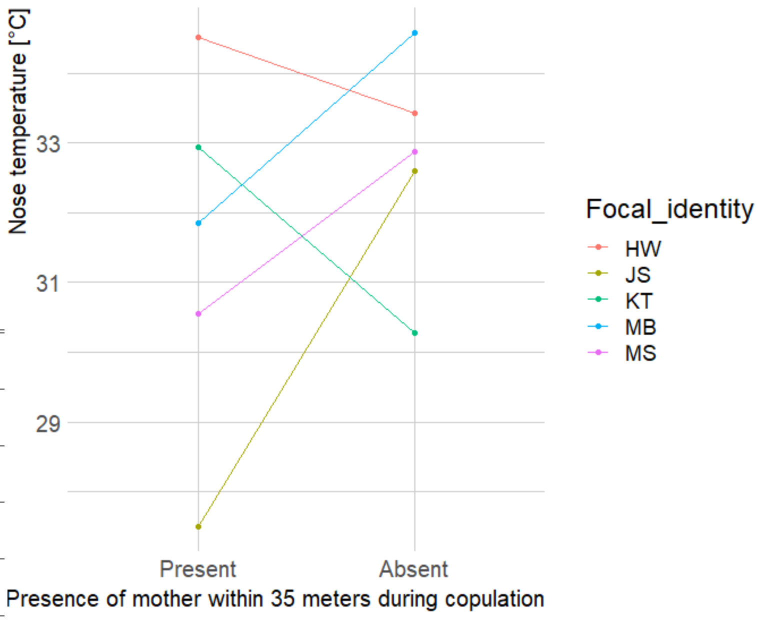

Supplementary Figure S2. Individual thermal response to the mother's presence during copulation. Even if the main model showed an overall effect of the presence of the mother, probably due to the consideration of males with no living mother, the sample is not big enough when considering only the individuals with living mothers (left). Moreover, two males (KT and HW) contribute with single observations to one or both conditions and should therefore be excluded or considered with caution (right).

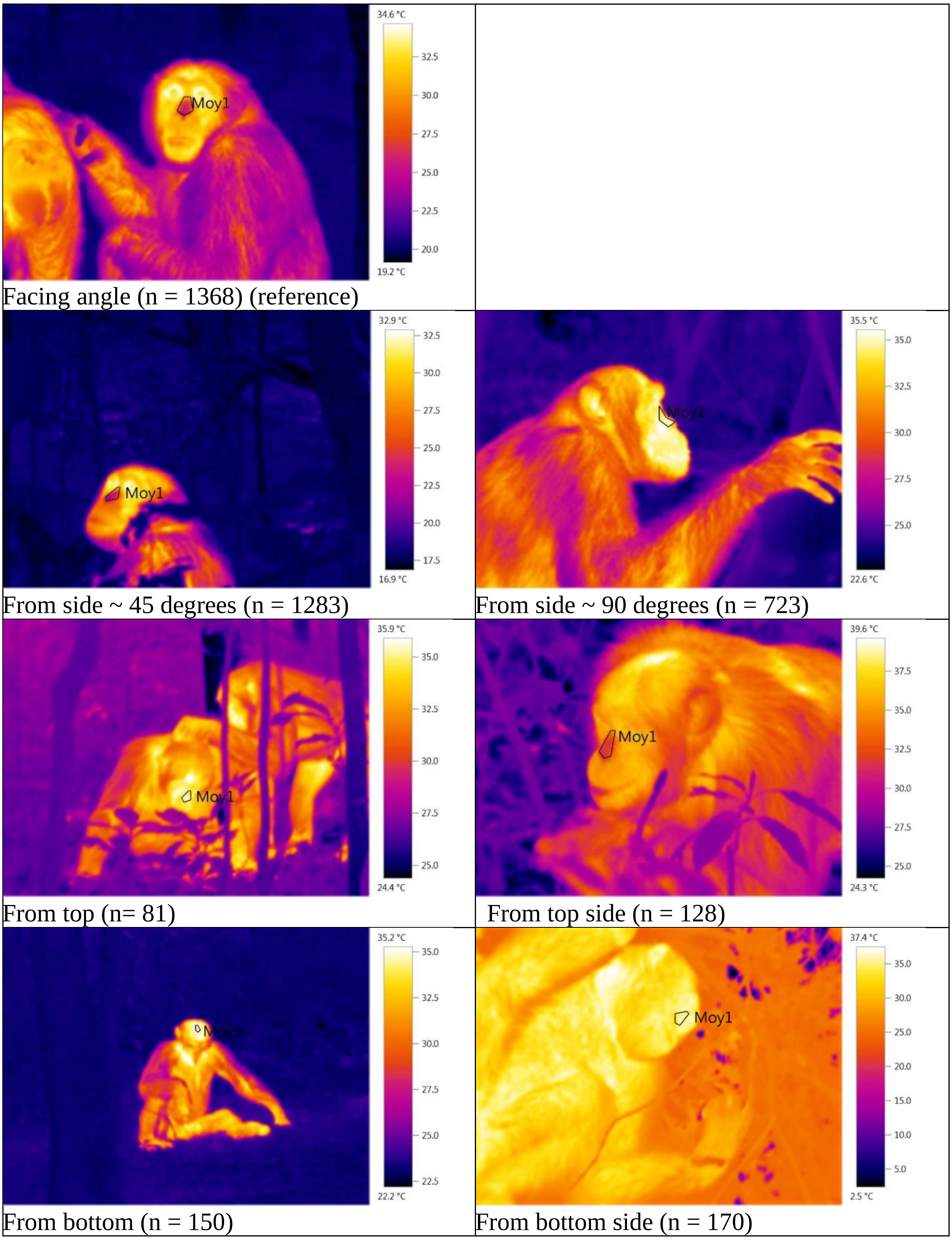

Supplementary Figure S3. The different angle categories used to categorise thermal pictures. Each picture was assigned within one of the 7 categories based on its visual aspect. The nose temperature measured showed a significant mean difference on the baseline pictures. Moy1 represents the region of interest.

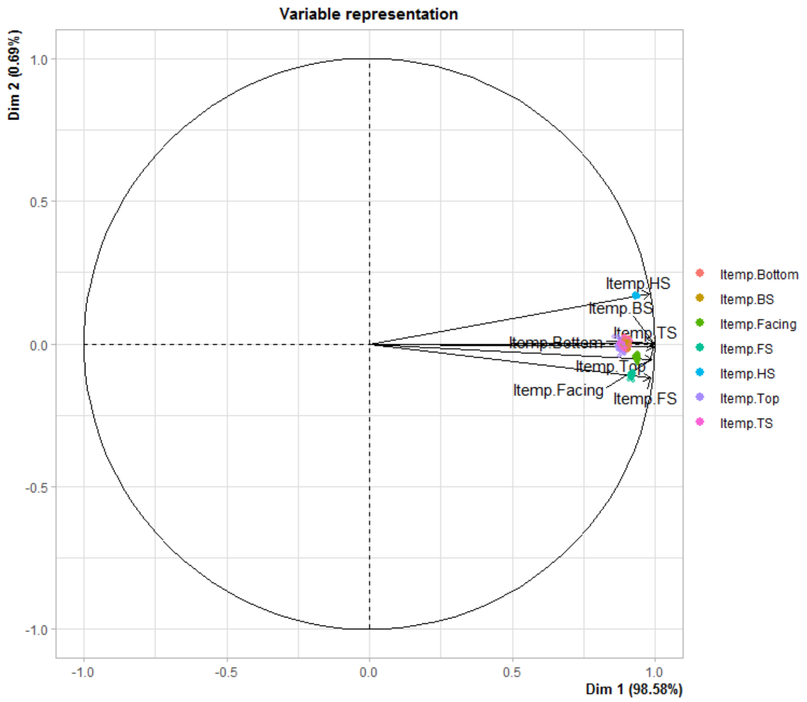

Supplementary Figure S4. Representation of a principal component analysis with missing data complemented. This was done after transforming the nose temperature with a logit function and using the *missMDA* R package.

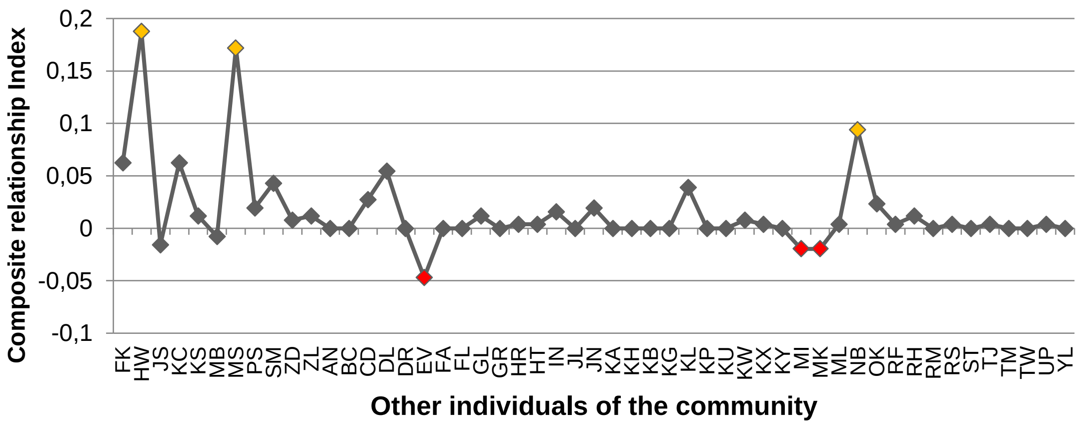

Supplementary Figure S5. Example of CRI index for the focal individual KT with all other individuals of the community. The three highest CRI scores are represented in yellow and determine the three social bond partners. The three lowest CRI scores define the social non-bond partners and are illustrated in red.

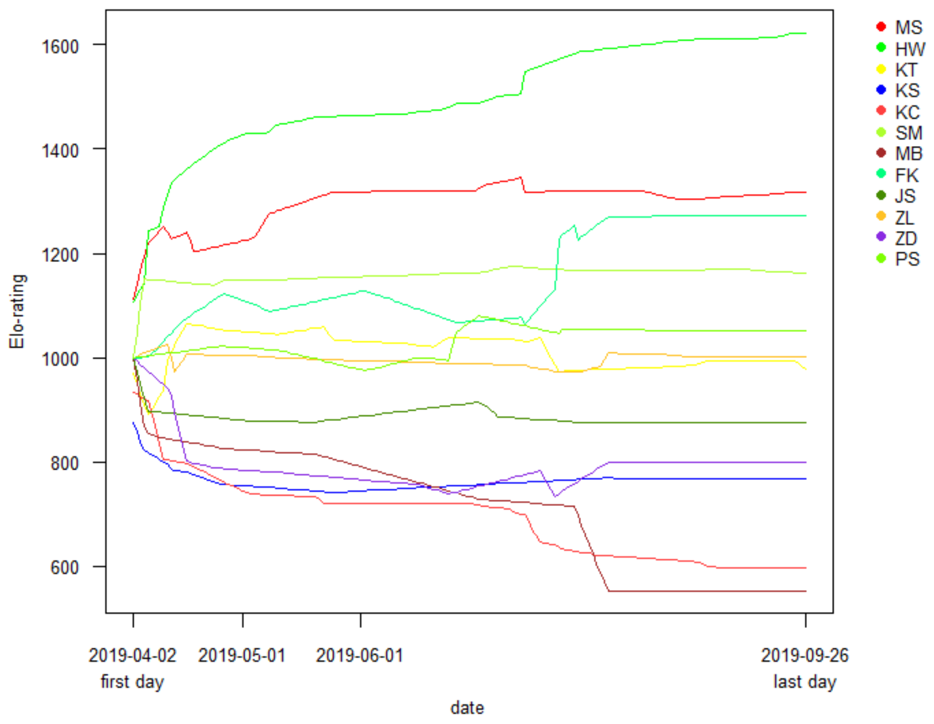

Supplementary Figure S6. The evolution of Elo-rating scores of male individuals used to extract their ranking score.

Data collected on male individuals (ID initials on the right) between 02.04.2019 (six months prior data collection) and 26.09.2019 (beginning of data collection). The score on the last day is used as the ranking score.

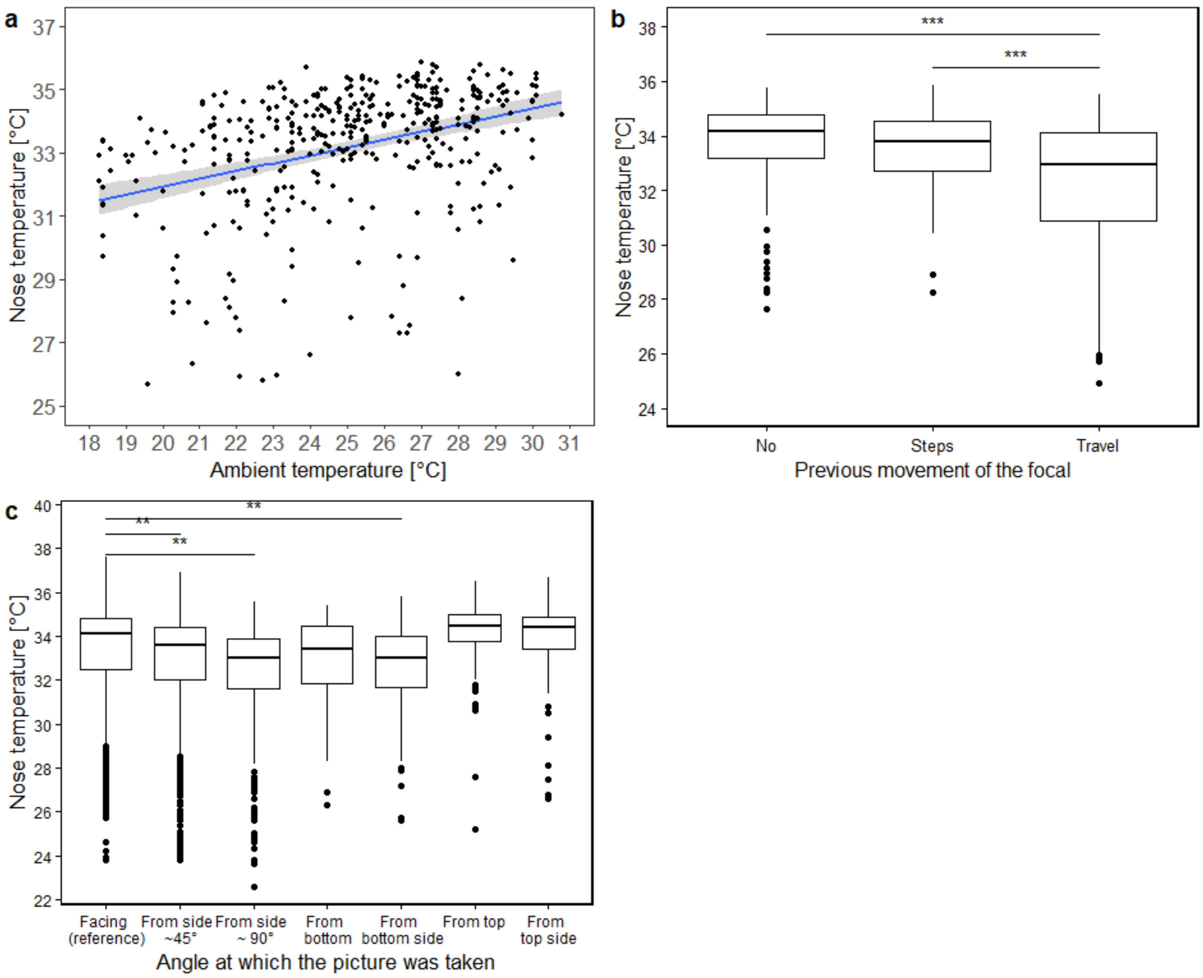

Supplementary Figure S7. The impact of three intervening variables on nose temperature of resting individuals. a. The impact of ambient temperature on resting chimpanzees' nose temperatures [°C]. (n = 392). **b.** Boxplots of nose temperatures by the previous movement of the chimpanzee, with *travel* (> 20 steps in the previous 5 minutes; M = 32.12, SD = 2.80) having a significantly lower temperature than *steps* (< 20 steps in 5 min; M = 33.47, SD = 1.47) (t(311) = -3.80, $p < .001$) and *no movement* (M = 33.71, SD = 1.68)) (t(310) = -7.07, $p < .001$). *Steps* was not but close to significantly different than *no movement* (t(309) = 2.27, $p = .062$). **c.** Boxplots of nose temperatures for all angle categories at which the thermal pictures were taken (see fig. S6) on resting chimpanzees. A post hoc Tukey test showed a decrease of temperature in the pictures taken at 45 degrees ($p < .001$), 90 degrees ($p < .001$), and taken from the bottom side ($p < .001$) when compared to the angle of reference (*facing*). (*** $p < .001$ ** $p <.01$ * $p < .05$).

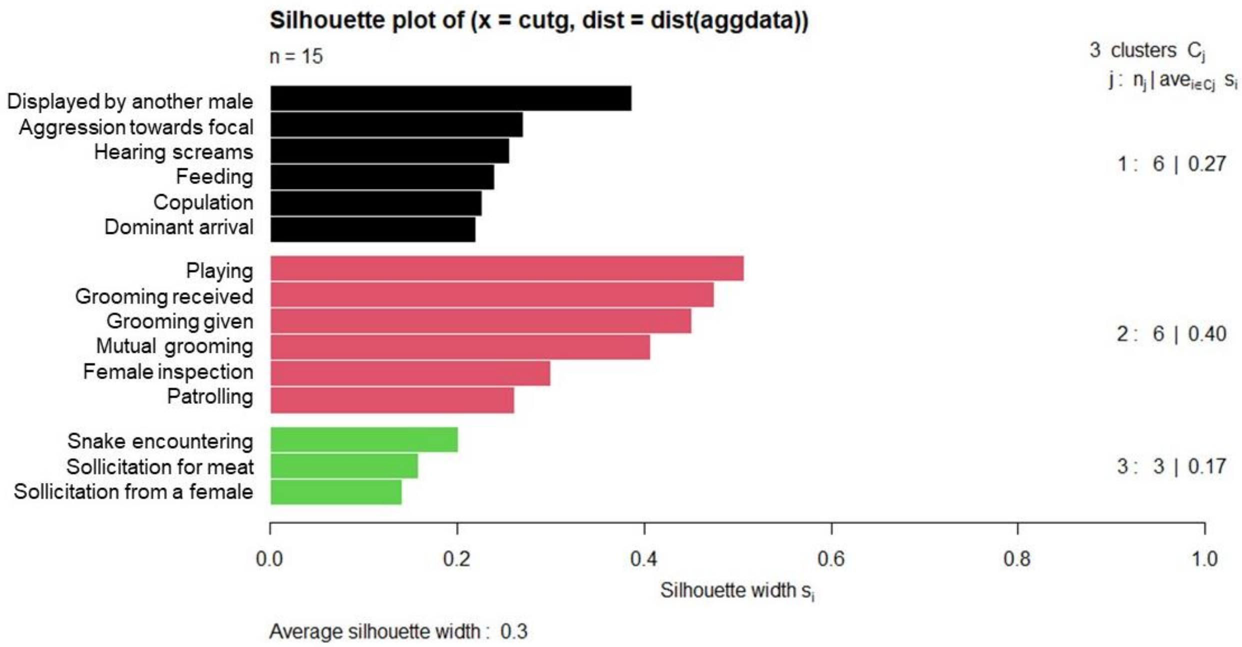

Supplementary Figure S8. Resulting silhouette plot for the cluster analysis, based on Ward's method.

SUPPLEMENTARY TABLES

Supplementary Table S1

Summary of studies that examined the link between negative or positive stimuli and temperature changes in the nasal region of different species.

	Authors	Species	Stimuli	Temperature change
**Negative stimuli**				
	Baker et al., 1976 [1]	Pigtail monkeys	Sudden illumination of the room, loud noises or the entrance of a human into the room	Decrease
	Brügger et al., 2021 [2]	Marmosets	Playback of aggressive calls	Decrease
	Chotard et al., 2018 [3]	Marmosets; capuchins; macaques; gibbons; gorillas	Food delay and teasing	Decrease
	Cruz-Albarran et al., 2017 [4]	Humans	Videos of tenebrous scenes	Decrease
	Dezecache et al., 2017 [5]	Chimpanzees	Hearing of aversive calls	Decrease
	Ermatinger et al., 2019 [6]	Common marmosets	Playback of aggressive vocalizations	Decrease
	Kano et al., 2016 [7]	Chimpanzees	Playback of fights	Decrease
	Kuraoka & Nakamura, 2011 [8]	Macaque monkeys	Threatening stimulus: video of a raging individual, facial expressions and voice of threatening calls	Decrease
	Merla & Romani, 2007 [9]	Humans	Stroop test with unknown person observing	Decrease
	Nakayama et al., 2005 [10]	Rhesus monkeys	Approach of threatening people	Decrease
	Or & Duffy, 2007 [11]	Humans	Cognitive workload	Decrease
**Positive stimuli**				
	Brügger et al., 2021 [2]	Marmosets	Playback of cooperative calls	Increase
	Chotard et al., 2018 [3]	Marmosets, capucins, macaques,	Interaction with toys and tickling	Decrease

		gibbons, gorillas		
	Cruz-Albarran et al., 2017 [4]	Humans	Funny videos	Decrease
	Ermatinger et al., 2019 [6]	Marmosets	Food and playback of food calls	Females: no change; Males: increase
	Grandi & Heinzl, 2016 [12]	Rhesus macaques	Sweep (pleasant touch)	Increase
	Hahn et al., 2012 [13]	Humans	Sexual arousal	Increase
	Heintz et al., 2019 [14]	Gorillas	Memory task	Mixed
	Ioannou et al., 2015 [15]	Rhesus macaques	Feeding	Decrease
	Ioannou et al., 2015 [15]	Rhesus macaques	Playing	Increase
	Kosonogov et al., 2017 [16]	Humans	Pleasant pictures	Decrease
	Nakanishi & Imai-Matsumura, 2008 [17]	Humans	Infants laughing	Decrease
	Proctor & Carder, 2015 [18]	Dairy cows	Stroking	Decrease
	Riemer et al., 2016 [19]	Dogs	Reunion after separation with human	Increase
	Salazar-López et al., 2015 [20]	Humans	Thinking about a beloved person or God for religious participants	Increase
	Tamioso et al., 2017 [21]	Sheep	Brushed by familiar observer	Increase

REFERENCES FOR SUPPLEMENTARY TABLE 1

1. Baker, M. A., Cronin, M. J. & Mountjoy, D. G. Variability of skin temperature in the waking monkey. *Am. J. Physiol.-Leg. Content* **230**, 449–455 (1976).
2. Brügger, R. K., Willems, E. P. & Burkart, J. M. Do marmosets understand others' conversations? A thermography approach. *Sci. Adv.* **7**, eabc8790 (2021).
3. Chotard, H., Ioannou, S. & Davila-Ross, M. Infrared thermal imaging: Positive and negative emotions modify the skin temperatures of monkey and ape faces. *Am. J. Primatol.* **80**, e22863 (2018).
4. Cruz-Albarran, I. A., Benitez-Rangel, J. P., Osornio-Rios, R. A. & Morales-Hernandez, L. A. Human emotions detection based on a smart-thermal system of thermographic images. *Infrared Phys. Technol.* **81**, 250–261 (2017).
5. Dezecache, G., Zuberbühler, K., Davila-Ross, M. & Dahl, C. D. Skin temperature changes in wild chimpanzees upon hearing vocalizations of conspecifics. *R. Soc. Open Sci.* **4**, 160816 (2017).
6. Ermatinger, F. A., Brügger, R. K. & Burkart, J. M. The use of infrared thermography to investigate emotions in common marmosets. *Physiol. Behav.* **211**, 112672 (2019).
7. Kano, F., Hirata, S., Deschner, T., Behringer, V. & Call, J. Nasal temperature drop in response to a playback of conspecific fights in chimpanzees: A thermo-imaging study. *Physiol. Behav.* **155**, 83–94 (2016).
8. Kuraoka, K. & Nakamura, K. The use of nasal skin temperature measurements in studying emotion in macaque monkeys. *Physiol. Behav.* **102**, 347–355 (2011).
9. Merla, A. & Romani, G. L. Thermal signatures of emotional arousal: a functional infrared imaging study. in *2007 29th Annual International Conference of the IEEE Engineering in Medicine and Biology Society* 247–249 (IEEE, 2007).
10. Nakayama, K., Goto, S., Kuraoka, K. & Nakamura, K. Decrease in nasal temperature of rhesus monkeys (Macaca mulatta) in negative emotional state. *Physiol. Behav.* **84**, 783–790 (2005).
11. Or, C. K. & Duffy, V. G. Development of a facial skin temperature-based methodology for non-intrusive mental workload measurement. *Occup. Ergon.* **7**, 83–94 (2007).

12. Grandi, L. C. & Heinzl, E. Data on thermal infrared imaging in laboratory non-human primates: Pleasant touch determines an increase in nasal skin temperature without affecting that of the eye lachrymal sites. *Data Brief* **9**, 536–539 (2016).
13. Hahn, A. C., Whitehead, R. D., Albrecht, M., Lefevre, C. E. & Perrett, D. I. Hot or not? Thermal reactions to social contact. *Biol. Lett.* **8**, 864–867 (2012).
14. Heintz, M. R., Fuller, G. & Allard, S. Exploratory investigation of infrared thermography for measuring gorilla emotional responses to interactions with familiar humans. *Animals* **9**, 604 (2019).
15. Ioannou, S., Chotard, H. & Davila-Ross, M. No strings attached: physiological monitoring of rhesus monkeys (Macaca mulatta) with thermal imaging. *Front. Behav. Neurosci.* **9**, 160 (2015).
16. Kosonogov, V. *et al.* Facial thermal variations: A new marker of emotional arousal. *PloS One* **12**, e0183592 (2017).
17. Nakanishi, R. & Imai-Matsumura, K. Facial skin temperature decreases in infants with joyful expression. *Infant Behav. Dev.* **31**, 137–144 (2008).
18. Proctor, H. S. & Carder, G. Nasal temperatures in dairy cows are influenced by positive emotional state. *Physiol. Behav.* **138**, 340–344 (2015).
19. Riemer, S., Assis, L., Pike, T. W. & Mills, D. S. Dynamic changes in ear temperature in relation to separation distress in dogs. *Physiol. Behav.* **167**, 86–91 (2016).
20. Salazar-López, E. *et al.* The mental and subjective skin: Emotion, empathy, feelings and thermography. *Conscious. Cogn.* **34**, 149–162 (2015).
21. Tamioso, P. R., Rucinque, D. S., Taconeli, C. A., da Silva, G. P. & Molento, C. F. M. Behavior and body surface temperature as welfare indicators in selected sheep regularly brushed by a familiar observer. *J. Vet. Behav.* **19**, 27–34 (2017).

Supplementary Table S2

List of all individuals from the Sonso community in February 2020.

Individual ID	Sex	Age category	Year of birth
**AC**	F	Infant	2017
**AN**	F	Adult	1990
**CD**	F	Young adult	2003
**DL**	F	Young adult	2002
**DB**	F	Infant	2018
**DR**	F	Young adult	2004
**ER**	F	Infant	2019
**EV**	F	Young adult	2004
**FA**	F	Sub-adult	2006
**FH**	F	Juvenile	2013
**FL**	F	Adult	1979
**FK**	M	Adult	1999
**GF**	M	Juvenile	2013
**GH**	F	Infant	2020
**GL**	F	Adult	1976
**HD**	M	Infant	2017
**HM**	F	Juvenile	2013
**HR**	F	Sub-adult	2009
**HT**	F	Adult	1978
**HW**	M	Adult	1993
**IN**	F	Adult	1999
**IS**	F	Infant	2017
**JA**	F	Infant	2018
**JB**	M	Juvenile	2011
**JL**	F	Adult	1990
**JN**	F	Adult	1984
**JS**	M	Sub-adult	2006
**KA**	F	Adult	1998
**KC**	M	Sub-adult	2006
**KH**	F	Sub-adult	2008
**KF**	M	Juvenile	2014
**KG**	F	Adult	1998
**KJ**	M	Juvenile	2013
**KL**	F	Adult	1979
**KO**	M	Juvenile	2014
**KQ**	M	Infant	2016
**KS**	M	Adult	2003
**KT**	M	Adult	1993
**KU**	F	Adult	1979
**KV**	M	Juvenile	2014
**KW**	F	Adult	1981
**KX**	F	Sub-adult	2007

**KY**	F	Adult	1983
**MB**	M	Sub-adult	2009
**MI**	F	Sub-adult	2007
**MK**	F	Adult	1980
**ML**	F	Adult	1975
**MS**	M	Adult	1992
**MZ**	M	Infant	2015
**NB**	F	Adult	1962
**OK**	F	Adult	1996
**OZ**	M	Juvenile	2014
**PS**	M	Adult	1998
**RF**	F	Sub-adult	2007
**RH**	F	Adult	1965
**RM**	F	Young adult	2002
**RS**	F	Adult	1997
**SM**	M	Adult	1993
**ST**	F	Sub-adult	2007
**TJ**	F	Adult	1984
**TW**	F	Sub-adult	2010
**UP**	F	Adult	1999
**ZD**	M	Young adult	2001
**ZL**	M	Adult	1995

Supplementary Table S3

List of focal individuals with respective age and rank as well as and number of separated recorded events of interest and baseline events. Rank hierarchy is based on Elo-rating score.

**Focal ID**	**Age**	**Rank**	**Number events**	**Number baselines**
FK	20	3	69	43
HW	26	1	129	49
JS	13	8	62	33
KT	26	7	38	42
KS	10	12	41	30
MS	27	2	89	56
PS	21	5	74	48
SM	26	4	70	64
ZL	24	6	39	27
Total			**611**	**392**

Supplementary Table S4

Results of the best model for cooperative events.

	Parameter estimate	Standard error	Degree of freedom	t value	p-value
**Intercept**	2.28	0.07	2.94	33.02	< 0.001
**Presence of alpha within 35 meters**	0.17	0.06	285.33	3.11	0.002
**Elo-rating score**	-0.04	0.03	286.79	-1.53	0.126
**Ambient temperature**	0.08	0.01	285.18	6.86	< 0.001

Note. The best model selected is *temperature ~ presence of the alpha within 35 meters + Elo-rating + ambient temperature + (1¦ focal identity) + (1 ¦previous movement).* Reference level for *presence of alpha within 35 meters* was “Absence”.

Supplementary Table S5

Results of the best model for grooming events.

	Parameter estimate	Standard error	Degree of freedom	t value	p-value
**Intercept**	2.34	0.06	4.06	38.84	< 0.001
**Type of grooming: Grooming partner is the alpha**	0.60	0.19	231.51	3.17	0.002
**Ambient temperature**	0.08	0.01	231.63	7.04	< 0.001

Note. The best model selected is *temperature ~ Type of grooming:Grooming partner is the alpha + ambient temperature + (1¦ focal identity) + (1 ¦previous movement).* Reference level for *grooming partner is the alpha* was “Yes” and for *type of grooming* was “grooming as a receiver”. T values for main effects comprised in interactions are omitted.

Supplementary Table S6

Results of best model for competitive events.

	Parameter estimate	Standard error	Degree of freedom	t value	p-value
**Intercept**	2.12	0.05	5.81	42.34	< 0.001
**Number of females within 10 meters**	0.07	0.03	160.67	2.24	0.027
**Ambient temperature**	0.08	0.01	203.40	7.43	< 0.001

Note. The best model selected is *temperature ~ Number of females within 10m + ambient temperature + (1¦ focal identity) + (1 ¦previous movement).*

Supplementary Table S7

Results of the best model for copulation events.

	Parameter estimate	Standard error	Degree of freedom	t value	p-value
**Intercept**	1.77	0.16	36.17	10.77	< 0.001
**Presence of mother within 35 meters**	0.30	0.17	40.74	1.79	0.082
**Number of males within 10 meters**	0.19	0.08	41.00	2.36	0.023
**Ambient temperature**	0.10	0.03	39.67	3.36	< 0.001

Note. The best model selected is *temperature ~ Presence of mother within 35m + number of males within 10m + ambient temperature + (1¦ focal identity) + (1 ¦previous movement).* Reference level for presence of mother within 35 meters is “Absence”.
